# The association between interactive health literacy and dietary behaviors among Chinese college students: a large-scale cross-sectional study

**DOI:** 10.3389/fpsyg.2024.1363885

**Published:** 2024-05-30

**Authors:** Chunxiao Ma, Zhongyu Ren, Zhenqi Chen, Caifu Li

**Affiliations:** ^1^School of Business Management, Liaoning Technical University, Huludao, China; ^2^Research Institute of Educational Economics and Administration, Shenyang Normal University, Shenyang, Liaoning, China; ^3^School of Physical Education, Southwest University, Chongqing, China; ^4^College of General Education, Guangxi Arts University, Nanning, China; ^5^College of Sports Science, Shenyang Normal University, Shenyang, China

**Keywords:** interactive health literacy, dietary behaviors, Chinese, college students, cross-sectional study

## Abstract

**Background:**

The association between health literacy and healthy dietary behaviors has been explored in the European population. However, there is currently no evidence available specifically pertaining to Chinese college students particularly for interactive health literacy.

**Aims:**

The objective of this study was to investigate the association between interactive health literacy (IHL) and dietary behaviors in Chinese college students.

**Methods:**

This study included 11,856 Chinese college students (mean age = 18.8 years, SD = 1.2 years). We defined nine healthy dietary behaviors as consumption of water, egg, milk and milk products, vegetables, fruit, red meat, soy and soy products, seafood, and sugar-sweetened beverages. For each food group, participants who met the criterion for being a regular consumer of the item were assigned a score of 1, and otherwise were assigned a score of 0. Thus, the dietary behaviors score ranged from 0 to 9, with higher scores indicating healthier dietary behaviors. We used the revised 28-item Chinese Adolescent Interactive Health Literacy Questionnaire (CAIHLQ) to evaluate IHL; a higher score on this scale indicates a greater health literacy. Multivariate logistic regression was used to analyze the association between IHL level and frequency of different numbers of dietary behaviors.

**Results:**

After adjusting for sex, age, annual family income, place of residence, father’s education level, and mother’s education level, there was a clear and significant positive association between IHL and the likelihood of exhibiting diverse dietary behaviors. The adjusted odds ratio (95% CI) of exhibiting given nine dietary behaviors with reference to tertile 1 according to categories of IHL was as follows: 1.055 (0.694, 1.603) for tertile 2 and 1.849 (1.269, 2.696) for tertile 3 (p for trend = 0.001). Similarly, there are significant positive associations between IHL and the likelihood of exhibiting 2–8 dietary behaviors, except for exhibiting any one dietary behavior. We further found that, in addition to the health awareness factor, there were significant positive associations between physical activity and nutrition factors, and healthier dietary behaviors. Further, there was a significant negative association between interpersonal relationships and dietary behavior.

**Conclusion:**

The findings indicate a positive relationship between IHL and dietary behavior, such that the higher the level of IHL among college students, the healthier the dietary behavior they tend to adopt in their daily lives. These findings suggest the importance of developing stages of change-based educational interventions, which could help individuals with limited IHL to not only acquire necessary health-related knowledge but also to strengthen their motivation to engage in healthy dietary behaviors. Future studies should employ longitudinal prospective designs or randomized controlled trials to establish a causal association between IHL and healthy dietary behaviors.

## 1 Introduction

Health literacy is defined as an ability that individual can obtain to understand and utilize health information and services, and make reasonable decisions to prevent diseases and promote health (Sorensen et al., 2012). Numerous epidemiological studies have indicated that health literacy varies greatly among countries and populations; compared with developed countries in Europe and America, Asian population have a higher proportion of low health literacy. For example, a recent meta-analysis of European Union member states indicated a prevalence of low health literacy from 27 to 48% (Baccolini et al., 2021). Similarly, an American population-based meta-analysis reported the prevalence of low health literacy was 26% (Paasche-Orlow et al., 2005). However, the proportion the population exhibiting low health literacy is up to 55.3% in southeast Asian countries (Rajah et al., 2019). The China Health Literacy Survey also indicated that only 23.15% of Chinese residents had adequate health literacy. Although health literacy has steadily advanced, from 6.48% in 2009 (Ministry of Health of the People’s Republic of China, 2009) to 23.15% in 2020 (National Health Commission of the People’s Republic of China, 2021), the Chinese government issued a “Healthy China Action Plan,” one goal of which is to reach 30% or more of Chinese residents demonstrating adequate health literacy by 2030 (Healthy China Action Promotion Committee, 2019). Based on the above findings, we suggest it is crucial that individuals possess good health literacy to maintain a healthy status.

Intervening in health literacy may improve people’s health behaviors because interventions can provide tools to translate knowledge into action and enable individuals to make healthy decisions currently and in the future. Among various health behaviors, dietary behavior has received increasing attention from researchers in recent years. The Lancet Collaboration conducted a systematic analysis based on data from 188 countries that compared the effects of 79 behavioral, environmental, and occupational factors on metabolic health. The findings showed that diet-related risks contribute the highest number of deaths and disability-adjusted life years when compared to any other risk factor globally, including smoking (Forouzanfar et al., 2015). This highlights the critical importance of improving human diets.

With the increase in the educational level and the phenomenon of late marriage and childbirth among modern people, a transition phase of “emerging adulthood” has been suggested, which is typically defined as ages 18–25 (Arnett, 2000). Emerging adulthood may be an important but overlooked period for establishing long-term healthy dietary behaviors due to identity development and constantly changing interpersonal relationships. For example, a longitudinal study of changes in dietary intake among the population aged 13–30 years found that after a decrease from 14 to 23 years and from 14 to 21 years, intake of vegetables and fruits increased to a peak at *circa* about 30 years, while the intake of sugary drinks and candies began to decrease after reaching a peak at *circa* about 18 years. The study also suggested that the later stage of adolescence is a critical window, as the trend in dietary habits at this time may continue into adult life (Winpenny et al., 2018). Subsequently, another longitudinal survey of individuals aged 30 to 59 years found that in a trajectory of “significant improvement,” overall Healthy Eating Index (HEI) scores shifted from poor/intermediate levels to good dietary categories as age increased. In a trajectory of “moderate improvement,” as age increased, the overall HEI score shifted from poor to moderate dietary categories, but the prevalence of good dietary categories remained low. This result also suggests that improving dietary habits in middle age may be beneficial for better health in later years (Talegawkar et al., 2021). Dietary habits early in life can adversely affect long-term health outcomes, including type 2 diabetes (Malik et al., 2012), hypertension (Sun Q. et al., 2022; Sun X. et al., 2022), colorectal cancer (Thordardottir et al., 2022), and arterial stiffness (Van de Laar et al., 2013). Prior to enrolling in college, students’ dietary behaviors are primarily influenced by their families and schools (Hayek et al., 2020). In contrast, the college phase offers students new experiences and personal freedom, including autonomy in food choices. However, this stage is also marked by the emergence of various unhealthy behaviors, thereby increasing the risk of health problems for college students (Fulkerson et al., 2004; GBD Diet Collaborators, 2019). Among the most prominent unhealthy dietary behaviors observed in college students are suboptimal in dietary habits (GBD Diet Collaborators, 2019).

Due to the importance of enhancing health literacy to minimize health risks, prevent diseases, and promote overall well-being, numerous researchers have examined the association between health literacy and health risk behaviors. One systematic review of 10,997 individuals across nine distinct countries revealed that health literacy interventions are effective at transforming health-related behaviors, specifically in terms of dietary preferences (Walters et al., 2020). A small number of researchers considered the relationship between diet and both programmed and declarative nutritional knowledge. Based on the perspective of public health, Nutbeam proposed that health literacy includes three levels: functional health literacy, interactive health literacy, and critical health literacy. Functional health literacy refers to the basic reading and writing skills and computational abilities that individuals need in their daily lives (Nutbeam, 2000). Given previous studies indicating that functional health literacy is an important predictor of healthy eating behaviors (Fleary et al., 2018), subsequent research focused on the relationship between functional health literacy and dietary behavior among Chinese adults, finding that individuals with greater health literacy were more likely to exhibit healthier eating patterns (Von Wagner et al., 2007; Guntzviller et al., 2017). This also suggests that health-related knowledge regarding food consumption requires priority attention, including improving individual abilities to access, understand, and apply health information. However, few studies have explored the relationship between interactive health literacy (IHL) and dietary behaviors among college students. A previous study of Chinese college students found the least literacy for “health lifestyle and behavior” (13.37%) compared to basic knowledge (41.77%) and concepts and basic skills (73.04%) (Guo Jing and Sha, 2011). This indicates that college students have a poor ability to apply health information to form or change healthy behaviors. The role of IHL—using the IHL scale to evaluate individuals’ capacity to access, understand, and apply health information—in the promotion of a healthy lifestyle and behavior among Chinese residents remains to be underexplored. In summary, this study collected data on the IHL and dietary behavior of college students in China to serve two objectives: (1) to assess IHL and diversity of dietary behaviors; (2) to examine IHL in subgroups with different diversity of dietary behaviors. This study offers guidance on identifying interventions that should be prioritized and target populations for healthy dietary intervention programs in Chinese college students.

## 2 Methods

### 2.1 Participants

This cross–sectional study investigated the protective and risk factors for physical fitness among Chinese college students. Data collection was conducted in October 2023. The survey sites of this study were based on annual physical fitness assessments at physical testing centers in 11 universities in China (6 universities in Liaoning Province, 1 in Jilin City, and 4 in Chongqing City). This study covered 33 majors including philosophy, logic, economics, finance, economics and trade, politics, sociology, preschool education, primary education, Chinese language and literature, foreign language and literature, history, mathematics, physics, psychology, statistics, engineering mechanics, mechanical design and manufacturing and automation, materials science and engineering, energy and power engineering, electronic science and technology, automation, management science, business management, accounting, human resource management, public management, tourism management, music and dance, drama, and art. Convenience sampling was utilized to select 1–2 grades of all students from each university in 33 majors as participants. The study involved a total of 11,856 participants. All participants were also required to complete a questionnaire that collected information on demographic variables, dietary behaviors and IHL. The sample size was determined through power calculations, employing the following formula:


$$n={\left(\frac{u_{a}}{\Delta }\right)}^{2}\times p\left(1-p\right)$$

According to data from the China Residents’ Dietary Guidelines Scientific Research Report (2021), over 50% of Chinese residents exhibit dietary imbalances. Based on these results, 461 was the minimum sample size for the current study.

Ethics approval was obtained from the Research Institute of Educational Economics and Administration of Shenyang Normal University (NO: RIEA-JYG-AM-047-2.0). All participants aged ≥16 years or their primary guardians of participants aged <16 years agreed to participate in this study and signed informed consent forms via WeChat and sent the informed consent form to the study authors in image format.

### 2.2 Assessment of dietary behaviors

Dietary behaviors were assessed based on the recommended Chinese Dietary Guidelines 2022 (Chinese Nutrition Society, 2022) or previous studies (Cai et al., 2021; Sun Q. et al., 2022; Sun X. et al., 2022). As recommended by the Chinese Dietary Guideline 2022, all participants were requested to report their dietary behaviors, which included their intake of water, eggs, milk and milk products, vegetables, fruit, red meat, soy and soy products, and seafood. An additional item for sugar-sweetened beverages was derived from a prospective cohort study that demonstrated that compared with individuals who consumed fewer than 1 cup of sugar-sweetened beverages per month, those who drank ≥2 cups of sugar-sweetened beverages daily were associated with a 21% higher risk of premature death (Singh et al., 2015). The quantity of water consumed was evaluated using the question: “How much water do you consume on average per day?” Six options were provided for participants to choose from: less than 250 mL, 250–500 mL, 500–1,000 mL, 1,000–1,500 mL, 1,500–2000 mL, and more than 2000 mL. Regarding water consumption, the participants were categorized as regular consumers (1,500 mL per day) and irregular consumers (less than 1,500 mL per day) (Chinese Nutrition Society, 2022). The quantity of sugar-sweetened beverages was evaluated by asking the question: “How many sugar-sweetened beverages do you consume on average per day?” Five options were provided for participants to choose from: none (never consume), less than 500 mL, 500–1,000 mL, 1,000–1,500 mL, and more than 1,500 mL. Regarding sugar-sweetened beverage consumption, the participants were categorized as consumers and non-consumers (never consume). The quantity of eggs consumed was evaluated by asking the question: “How many eggs do you eat on average per day?” Five options were provided for participants to choose from: none (never consume), one, two, three, four or more. Regarding egg consumption, the participants were categorized as regular consumers (at least one per day) and irregular consumers (never consume). The quantity of milk and milk products was evaluated by asking the question: “How much milk and milk products do you consume on average per day?” Five options were provided for participants to choose from: none (never consume), less than 250 mL, 250–500 mL, 500–750 mL, and more than 750 mL. Regarding milk consumption, the participants were categorized as regular consumers (at least 250 mL per day) and irregular consumers (less than 250 mL or never consume). The consumption of vegetables or fruits was evaluated by asking the question: “How frequently do you consume vegetables or fruits on average per day?” Five options were provided for participants to choose from: never, once, twice, three times, and more than four times. Regarding vegetable or fruit consumption, the participants were categorized as regular consumers (at least once per day) and irregular consumers (never). The participants were also asked “How frequently do you consume red meat (including pork, beef, lamb, and processed products such as bacon and sausages) or soy products or seafood on average per week?” The eight options ranged from never eating to eating daily. Regarding red meat, soy products, or seafood consumption, the participants were categorized as regular consumers (at least twice per week) and irregular consumers (less than twice per week). For each food group, participants who met the criterion for being a consumer of the item were assigned a score of 1, and otherwise were assigned a score of 0. Thus, the dietary behaviors score ranged from 0 to 9, with higher scores indicating healthier dietary behaviors (Zhu et al., 2019). The detailed questions and options regarding dietary behaviors are presented in Table 1. Intraclass correlation coefficient (ICC) was employed to assess the test–retest reliability of the aforementioned dietary behaviors within 5 months. The ICC values were found to be statistically significant and ranged from 0.947 to 0.987 indicating that these dietary habits possessed excellent test–retest reliability.

**Table 1:** Description of each item and option for dietary behavior.

Item	Questions and options	
Quantity of water	Question	How much water do you consume on average per day?
	Options	□ Less than 250 mL □ 250-500ml □ 500-1000ml □ 1000-1500ml □ 1500-2000ml □ More than 2000 mL
Quantity of egg	Question	How many eggs do you eat on average per day?
	Options	□ Never □ One egg □ Two eggs □ Three eggs □ Four or more eggs
Quantity of milk and milk products	Question	How much milk and milk products do you consume on average per day?
	Options	□ never □ less than 250 mL □ 250-500ml □ 500-7500ml □ more than 750 mL
Quantity of vegetables	Question	How frequency do you consume vegetables on average per day?
	Options	□ Never □ Once □ Twice □ Three times □ More than four times
Quantity of fruits	Question	How frequency do you consume fruits on average per day?
	Options	□ Never □ Once □ Twice □ Three times □ More than four times
Quantity of red meat	Question	How frequency do you consume red meat (including pork, beef, lamb, and processed products such as bacon and sausages) on average per week?
	Options	□ Never □ Once □ Twice □ Three times □ Four times □ Five times □ Six times □ Every day
Quantity of soy and soy products	Question	How frequency do you consume soy products on average per week?
	Options	□ Never □ Once □ Twice □ Three times □ Four times □ Five times □ Six times □ Every day
Quantity of seafood	Question	How frequency do you consume seafood on average per week?
	Options	□ Never □ Once □ Twice □ Three times □ Four times □ Five times □ Six times □ Every day
Quantity of sugar-sweetened beverages	Question	How many sugar-sweetened beverages do you consume on average per day?
	Options	□ Never □ Less than 500 mL □ 500-1000ml □ 1000-1500ml □ More than 1,500 mL

### 2.3 Assessment of interactive health literacy

The Chinese Adolescent Interactive Health Literacy Questionnaire (CAIHLQ) was used to evaluate IHL in this study. The reliability and validity of the CAIHLQ have been established as good in Chinese adolescent (Zhang et al., 2014). This original scale which incorporated transtheoretical model into the design of an CAIHLQ by setting items based on temporal dimensions and scoring methods for each stage of health behaviors including pre-contemplation, contemplation, preparation, action, and maintenance, consists of 31 items and encompasses six dimensions: physical activity, interpersonal relationships, stress management, self-actualization, health awareness, and dietary behavior. To examine the structural validity of this scale among Chinese college students, we conducted exploratory factor analysis with varimax rotation. The revised scale consisted of four dimensions and 28 items. The cumulative variance explained by these dimensions was 70.02%. The four factors were health awareness (factor 1), interpersonal relationships (factor 2), nutrition (factor 3) and physical activity (factor 4). Pearson correlations between each item and the total score ranged from 0.446 to 0.811, all of which were statistically significant, indicating that each factor had good discriminant validity. For each item, there are following five respond options: (1) Have not done or do not want to do it within the past 6 months; (2) Have not done but want to do it within the past 6 months; (3) Did it within the past month without consistency; (4) Did it regularly within the past 6 months; (5) Have been doing it consistently for more than 6 months. Each option is assigned a score of 1–5 points. To calculate the IHL score, we summed the scores of all items. A higher score on this scale indicated a higher level of IHL. The internal consistency reliability of the scale was confirmed by a Cronbach’s α coefficient of 0.956, indicating good reliability. The distribution of IHL scores was used to divide the study population into tertiles, allowing for comparisons between different levels of IHL. The detailed items and options regarding IHL are presented in Table 2.

**Table 2:** Factor analysis results and component analysis of the CAIHLQ.

Item	Components			
	Health awareness	Interpersonal relationship	Nutrition	Physical activities
1. Pay attention to regulating oneself in daily life and prevent fatigue	0.742			
2. Be able to balance study and leisure time	0.739			
3. Feel very meaningful every day	0.729			
4. Watch promotional and television programs about improving health	0.714			
5. Be brave enough to challenge new things	0.708			
6. Do things to relieve stress in daily life	0.697			
7. Strive towards the life goals	0.693			
8. Think some pleasant things before falling asleep every day	0.685			
9. Be full of hope for the future	0.671			
10. Ensure adequate sleep	0.604			
11. Look at the nutritional composition table on the food packaging bag	0.485			
12. Do not drink soft drinks (such as carbonated drinks, fruit juice drinks, natural mineral water drinks, and tea drinks)	0.469			
13. Be able to maintain good interpersonal relationships with classmates		0.868		
14. Care, love, and warm others regularly		0.845		
15. Be able to get along well with families		0.832		
16. Can receive help from others when facing difficulties		0.808		
17. Take time to be with family or friends		0.799		
18. Relax for 15–20 min per day		0.691		
19. Eat 200–400 g of vegetables per day			0.798	
20. Eat 250–400 g of grains per day			0.796	
21. Eat 200–400 g of fresh fruits per day			0.759	
22. Drink 250 mL of milk products per day			0.745	
23. Eat 80–110 g of red meat per day			0.724	
24. Do 60 min of moderate intensity exercise per day				0.866
25. Do 60 min of high-intensity exercise per day				0.854
26. Integrate sports into daily life				0.815
27. Take physical exercise in a planned way				0.770
28. Participate in entertainment sports projects				0.738

### 2.4 Assessment of covariates

All participants were also asked to complete a self-designed questionnaire. The questionnaire assessed demographic variables (gender, age), annual family income (<20,000, 20,000–35,000, >35,000 yuan), place of residence (town or rural), and father and mother’s education (primary and below, junior high, high school, or bachelor degree and above).

### 2.5 Statistical analysis

All statistical analyses were conducted using SPSS 24.0 (SPSS Inc., Chicago, IL, United States). Our analytic sample was defined as those who had complete data across all study variables. Continuous variables were presented as medians (interquartile range [IQR]) due to the non-normal distribution of the continuous variables, and categorical variables were presented as proportions. The differences in participant characteristics between participants with and without healthy dietary habits were assessed using kruskal-wallis test for continuous variables and Chi-square tests for categorical variables.

Meeting various dietary behaviors (coded as either ‘yes’ or ‘no’) was used as the dependent variable and IHL level tertiles as the independent variable. Multivariate logistic regression analyses were performed to examine the association between IHL level tertiles and healthy dietary behaviors. Model 1 was the raw model, whereas Model 2 adjusted for gender, age, annual family income, place of residence, father’s education, and mother’s education. We used multivariate adjusted models to explore the association between different dimensions (health awareness, physical activity, nutrition and interpersonal relationships) of IHL and dietary behaviors. Statistical significance as defined as *p* < 0.05, two-tailed.

## 3 Results

Table 3 shows the participants’ characteristics. This study comprised 11,856 college students (33.2% male) with a median age of 19.0 years, 58.9% of whom lived in rural area, with more than one-third (34.6%) reporting an annual household income exceeding 35,000 yuan. Only approximately one-third of the students had parents with a bachelor degree and above.

**Table 3:** Participants’ characteristics according to categories of dietary behaviors score^a^ (*n* = 11,856).

*N* = 11,856	Total	Categories of dietary habits (score)										
		0	1	2	3	4	5	6	7	8	9	*p*- value^b^
Sex (male), %	33.2	44.8	35.7	33.9	32.3	32.2	31.6	32.9	35.0	34.2	43.8	0.016
Age, years	19.0 (18.0, 19.0)	19.0 (18.0, 19.0)	19.0 (18.0, 20.0)	19.0 (18.0, 19.0)	19.0 (18.0, 19.0)	19.0 (18.0, 19.0)	19.0 (18.0, 19.0)	19.0 (18.0, 19.0)	19.0 (18.0, 19.0)	19.0 (18.0, 19.0)	19.0 (18.0, 19.0)	0.473
**Annual family income, yuan**												
<20,000	35.5 (4213)	50.0	33.9	37.0	38.6	38.5	36.9	34.7	32.8	30.6	29.0	<0.001
20,000–35,000	29.9 (3542)	20.7	23.2	32.8	30.4	31.4	29.0	29.5	29.0	32.2	27.2	
>35,000	34.6 (4101)	29.3	42.9	30.2	31.0	30.1	34.1	35.8	38.1	37.3	43.8	
**The place of residence, %**												
Town	41.1 (4875)	46.6	37.5	36.2	37.3	39.2	39.4	41.2	44.3	46.5	53.8	<0.001
Rural	58.9 (6981)	53.4	62.5	63.8	62.7	60.8	60.6	58.8	55.7	53.5	46.2	
**Father’s education level, %**												
Primary and below	23.9 (2833)	19.0	29.5	31.5	26.6	24.8	24.2	23.3	22.1	21.7	16.6	<0.001
Junior High	39.5 (4688)	60.3	43.8	40.3	39.6	40.5	39.9	40.1	38.1	35.7	41.4	
High School	21.4 (2541)	13.8	16.1	17.1	21.5	21.0	21.0	21.5	22.4	24.0	20.7	
Bachelor degree and above	15.1 (1794)	6.9	10.7	11.1	12.3	13.7	14.9	15.1	17.4	18.6	21.3	
**Mother’s education level, %**												
Primary and below	31.9 (3784)	27.6	39.3	42.4	35.5	34.0	31.4	31.2	29.1	29.1	27.8	<0.001
Junior High	36.4 (4314)	50.0	28.6	34.6	36.7	36.9	38.3	36.5	35.1	33.6	32.5	
High School	18.6 (2207)	13.8	19.6	13.2	17.3	17.7	17.5	19.9	20.4	20.1	15.4	
Bachelor degree and above	13.1 (1551)	8.6	12.5	9.8	10.5	11.4	12.7	12.4	15.4	17.1	24.3	

a means Continuous variables are expressed as medians (interquartile range) and categorical variables are expressed as percentages. b means *P* value were assessed using Kruskal Wallis test for continuous variables and chi-square test for categorical variables.

Table 3 also shows the comparison of participants’ characteristics across categories of dietary behaviors scores. The higher-score categories contained a higher proportion of males (*p* = 0.016). Individuals with a higher dietary behaviors score tended to have higher annual household income (*p* < 0.001), as did their parents (*p* < 0.001). Additionally, individuals with a higher dietary behaviors score were more likely to live in urban areas (*p* < 0.001).

**Figure 1:**
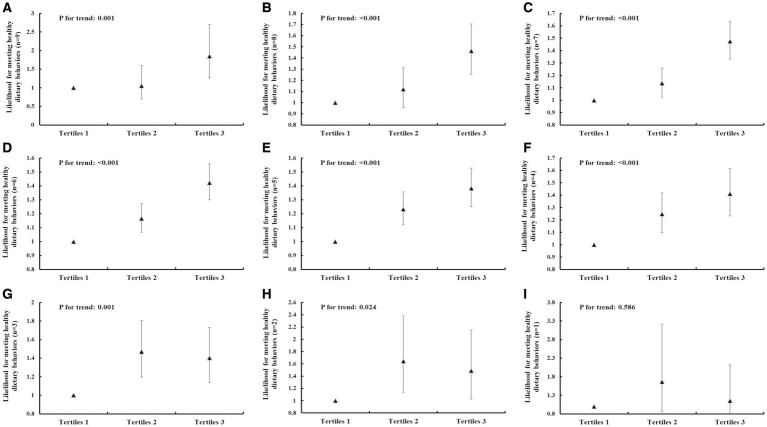
The association between IHL and meeting different numbers of healthy dietary behaviors. **(A)** Meeting nine healthy dietary behaviors. **(B)** Meeting eight healthy dietary behaviors. **(C)** Meeting seven healthy dietary behaviors. **(D)** Meeting six healthy dietary behaviors. **(E)** Meeting five healthy dietary behaviors. **(F)** Meeting four healthy dietary behaviors. **(G)** Meeting three healthy dietary behaviors. **(H)** Meeting two healthy dietary behaviors. **(I)** Meeting one healthy dietary behaviors. The data were exhibited as odds ratios (OR) and their corresponding 95% confidence intervals (CI). Logistic regression analysis was used to calculate *p* values. Adjusted for sex, age (continuous variable), annual family income (<20,000, 20,000–35,000, >35,000 yuan), the place of residence, father’s education level, mother’s education level. The horizontal axis represents the tertiles of IHL.

The results of the multivariate logistic regression analysis for identifying the independent associations between IHL and dietary behaviors score are shown in Figure 1. After adjusting for sex, age, annual family income, place of residence, father’s education level, and mother’s education level, there was a clear and significant positive association between IHL and the likelihood of exhibiting nine dietary behaviors. The adjusted odds ratio (95% CI) of meeting nine dietary behaviors for the categories of IHL, using tertile 1 as a reference, was 1.055 (0.694, 1.603) for tertile 2 and 1.849 (1.269, 2.696) for tertile 3 (p for trend = 0.001); for exhibiting eight dietary behaviors, the odds ratios were 1.121 (0.957, 1.312) and 1.463 (1.255, 1.705), respectively (*p* < 0.001); for seven behaviors the odds ratios were 1.137 (1.025, 1.260) and 1.474 (1.331, 1.633), respectively (*p* < 0.001); for six behaviors, the odds ratios were 1.166 (1.067, 1.273) and 1.423 (1.300, 1.558), respectively (*p* < 0.001); for five behaviors, the odds ratios were 1.232 (1.120, 1.356) and 1.382 (1.251, 1.527), respectively (*p* < 0.001); for four behaviors, the odds ratios were 1.248 (1.098, 1.419) and 1.411 (1.232, 1.615), respectively (*p* < 0.001); for three behaviors, the odds ratios were 1.470 (1.197, 1.806) and 1.403 (1.137, 1.730), respectively (*p* = 0.001); for two behaviors, the odds ratios were 1.646 (1.136, 2.384) and 1.487 (1.027, 2.153), respectively (*p* = 0.024); and for one behavior, the odds ratios were 1.662 (0.859, 3.217) and 1.155 (0.630, 2.118), respectively (*p* = 0.586). The adjusted odds ratio (95% CI) of meeting healthy water consumption frequency for the categories of IHL, using tertile 1 as a reference, was 1.056 (0.927, 1.203) for tertile 2 and 1.527 (1.347, 1.730) for tertile 3 (*p* for trend <0.001). The adjusted odds ratio (95% CI) of meeting healthy sugar-sweetened beverages consumption frequency for the categories of IHL, using tertile 1 as a reference, was 1.087 (0.986, 1.198) for tertile 2 and 0.925 (0.839, 1.020) for tertile 3 (*p* for trend = 0.128). The adjusted odds ratio (95% CI) of meeting healthy egg consumption frequency for the categories of IHL, using tertile 1 as a reference, was 1.176 (1.077, 1.285) for tertile 2 and 1.348 (1.231, 1.476) for tertile 3 (*p* for trend <0.001). The adjusted odds ratio (95% CI) of meeting healthy milk and milk products consumption frequency for the categories of IHL, using tertile 1 as a reference, was 1.042 (0.940, 1.154) for tertile 2 and 1.255 (1.133, 1.390) for tertile 3 (*p* for trend <0.001). The adjusted odds ratio (95% CI) of meeting healthy vegetables consumption frequency for the categories of IHL, using tertile 1 as a reference, was 1.359 (1.122, 1.647) for tertile 2 and 1.379 (1.131, 1.681) for tertile 3 (*p* for trend = 0.001). The adjusted odds ratio (95% CI) of meeting healthy fruits consumption frequency for the categories of IHL, using tertile 1 as a reference, was 1.222 (1.095, 1.363) for tertile 2 and 1.330 (1.187, 1.491) for tertile 3 (*p* for trend <0.001). The adjusted odds ratio (95% CI) of meeting healthy red meat consumption frequency for the categories of IHL, using tertile 1 as a reference, was 1.142 (1.021, 1.276) for tertile 2 and 1.260 (1.121, 1.415) for tertile 3 (*p* for trend <0.001). The adjusted odds ratio (95% CI) of meeting healthy soy and soy products consumption frequency for the categories of IHL, using tertile 1 as a reference, was 1.172 (1.062, 1.294) for tertile 2 and 1.378 (1.241, 1.529) for tertile 3 (*p* for trend <0.001). The adjusted odds ratio (95% CI) of meeting healthy seafood consumption frequency for the categories of IHL, using tertile 1 as a reference, was 0.987 (0.901, 1.080) for tertile 2 and 1.092 (0.996, 1.197) for tertile 3 (*p* for trend = 0.064) (Tables 4–12).

**Table 4:** The associations between IHL and water consumption (≥1,500 mL per day) (*n* = 11,856).

Categories of IHL	Total numbers	Model 1^a^	Model 2^b^
Tertiles 1	4,036	Reference	Reference
Tertiles 2	4,053	1.030 (0.906, 1.172)^c^	1.056 (0.927, 1.203)
Tertiles 3	3,767	1.542 (1.363, 1.744)	1.527 (1.347, 1.730)
P for trend^d^	—	<0.001	<0.001

a Model 1: crude.

b Model 2: Adjusted for sex, age (continuous variable), annual family income (<20,000, 20,000–35,000, >35,000 yuan), place of residence (town or rural), and father and mother’s education (primary and below, junior high, high school, or bachelor degree and above).

c Adjusted data are expressed as odds ratio (95% confidence intervals).

d P for trend were obtained using multivariate logistic regression analyses.

**Table 5:** The associations between IHL and sugar-sweetened beverages consumption (never consume) (*n* = 11,856).

Categories of IHL	Total numbers	Model 1^a^	Model 2^b^
Tertiles 1	4,036	Reference	Reference
Tertiles 2	4,053	1.079 (0.980, 1.189)^c^	1.087 (0.986, 1.198)
Tertiles 3	3,767	0.918 (0.833, 1.011)	0.925 (0.839, 1.020)
P for trend^d^	—	0.091	0.128

a Model 1: crude.

b Model 2: Adjusted for sex, age (continuous variable), annual family income (<20,000, 20,000–35,000, >35,000 yuan), place of residence (town or rural), and father and mother’s education (primary and below, junior high, high school, or bachelor degree and above).

c Adjusted data are expressed as odds ratio (95% confidence intervals).

d P for trend were obtained using multivariate logistic regression analyses.

**Table 6:** The associations between IHL and egg consumption (at least one per day) (*n* = 11,856).

Categories of IHL	Total numbers	Model 1^a^	Model 2^b^
Tertiles 1	4,036	Reference	Reference
Tertiles 2	4,053	1.185 (1.086, 1.294)^c^	1.176 (1.077, 1.285)
Tertiles 3	3,767	1.386 (1.267, 1.516)	1.348 (1.231, 1.476)
P for trend^d^	—	<0.001	<0.001

a Model 1: crude.

b Model 2: Adjusted for sex, age (continuous variable), annual family income (<20,000, 20,000–35,000, >35,000 yuan), place of residence (town or rural), and father and mother’s education (primary and below, junior high, high school, or bachelor degree and above).

c Adjusted data are expressed as odds ratio (95% confidence intervals).

d P for trend were obtained using multivariate logistic regression analyses.

**Table 7:** The associations between IHL and milk and milk products consumption (at least 250 mL per day) (*n* = 11,856).

Categories of IHL	Total numbers	Model 1^a^	Model 2^b^
Tertiles 1	4,036	Reference	Reference
Tertiles 2	4,053	1.049 (0.948, 1.162)^c^	1.042 (0.940, 1.154)
Tertiles 3	3,767	1.296 (1.172, 1.434)	1.255 (1.133, 1.390)
P for trend^d^	—	<0.001	<0.001

a Model 1: crude.

b Model 2: Adjusted for sex, age (continuous variable), annual family income (<20,000, 20,000–35,000, >35,000 yuan), place of residence (town or rural), and father and mother’s education (primary and below, junior high, high school, or bachelor degree and above).

c Adjusted data are expressed as odds ratio (95% confidence intervals).

d P for trend were obtained using multivariate logistic regression analyses.

**Table 8:** The associations between IHL and vegetable consumption (at least once per day) (*n* = 11,856).

Categories of IHL	Total numbers	Model 1^a^	Model 2^b^
Tertiles 1	4,036	Reference	Reference
Tertiles 2	4,053	1.361 (1.124, 1.646)^c^	1.359 (1.122, 1.647)
Tertiles 3	3,767	1.394 (1.146, 1.696)	1.379 (1.131, 1.681)
P for trend^d^	—	0.001	0.001

a Model 1: crude.

b Model 2: Adjusted for sex, age (continuous variable), annual family income (<20,000, 20,000–35,000, >35,000 yuan), place of residence (town or rural), and father and mother’s education (primary and below, junior high, high school, or bachelor degree and above).

c Adjusted data are expressed as odds ratio (95% confidence intervals).

d P for trend were obtained using multivariate logistic regression analyses.

**Table 9:** The associations between IHL and fruits consumption (at least once per day) (*n* = 11,856).

Categories of IHL	Total numbers	Model 1^a^	Model 2^b^
Tertiles 1	4,036	Reference	Reference
Tertiles 2	4,053	1.254 (1.125, 1.397)^c^	1.222 (1.095, 1.363)
Tertiles 3	3,767	1.367 (1.221, 1.529)	1.330 (1.187, 1.491)
P for trend^d^	—	<0.001	<0.001

a Model 1: crude.

b Model 2: Adjusted for sex, age (continuous variable), annual family income (<20,000, 20,000–35,000, >35,000 yuan), place of residence (town or rural), and father and mother’s education (primary and below, junior high, high school, or bachelor degree and above).

c Adjusted data are expressed as odds ratio (95% confidence intervals).

d P for trend were obtained using multivariate logistic regression analyses.

**Table 10:** The associations between IHL and red meat consumption (at least twice per week) (*n* = 11,856).

Categories of IHL	Total numbers	Model 1^a^	Model 2^b^
Tertiles 1	4,036	Reference	Reference
Tertiles 2	4,053	1.170 (1.048, 1.307)^c^	1.142 (1.021, 1.276)
Tertiles 3	3,767	1.318 (1.174, 1.478)	1.260 (1.121, 1.415)
P for trend^d^	—	<0.001	<0.001

a Model 1: crude.

b Model 2: Adjusted for sex, age (continuous variable), annual family income (<20,000, 20,000–35,000, >35,000 yuan), place of residence (town or rural), and father and mother’s education (primary and below, junior high, high school, or bachelor degree and above).

c Adjusted data are expressed as odds ratio (95% confidence intervals).

d P for trend were obtained using multivariate logistic regression analyses.

**Table 11:** The associations between IHL and soy and soy products consumption (at least twice per week) (*n* = 11,856).

Categories of IHL	Total numbers	Model 1^a^	Model 2^b^
Tertiles 1	4,036	Reference	Reference
Tertiles 2	4,053	1.193 (1.081, 1.317)^c^	1.172 (1.062, 1.294)
Tertiles 3	3,767	1.428 (1.288, 1.582)	1.378 (1.241, 1.529)
P for trend^d^	—	<0.001	<0.001

a Model 1: crude.

b Model 2: Adjusted for sex, age (continuous variable), annual family income (<20,000, 20,000–35,000, >35,000 yuan), place of residence (town or rural), and father and mother’s education (primary and below, junior high, high school, or bachelor degree and above).

c Adjusted data are expressed as odds ratio (95% confidence intervals).

d P for trend were obtained using multivariate logistic regression analyses.

**Table 12:** The associations between IHL and seafood consumption (at least twice per week) (*n* = 11,856).

Categories of IHL	Total numbers	Model 1^a^	Model 2^b^
Tertiles 1	4,036	Reference	Reference
Tertiles 2	4,053	1.005 (0.919, 1.099)^c^	0.987 (0.901, 1.080)
Tertiles 3	3,767	1.132 (1.034, 1.239)	1.092 (0.996, 1.197)
P for trend^d^	—	0.008	0.064

a Model 1: crude.

b Model 2: Adjusted for sex, age (continuous variable), annual family income (<20,000, 20,000–35,000, >35,000 yuan), place of residence (town or rural), and father and mother’s education (primary and below, junior high, high school, or bachelor degree and above).

c Adjusted data are expressed as odds ratio (95% confidence intervals).

d P for trend were obtained using multivariate logistic regression analyses.

## 4 Discussion

This cross-sectional study of a young population investigated the association between IHL level and the likelihood of exhibiting healthy dietary behaviors. The findings revealed that individuals with higher IHL levels were more likely to exhibit healthier dietary behaviors, even after accounting for potential confounding variables.

The findings of this study support earlier research indicating a positive association between general health literacy and the likelihood of exhibiting healthy dietary behaviors. To date, research on the association between health literacy and dietary behavior has largely been limited to cross-sectional studies conducted in Europe. In three cross-sectional studies in Turkey, regression analysis revealed that health literacy level independent predicted positive eating behavior, Furthermore, significant statistical associations were found between health literacy and the avoidance of high-fat diets and adoption of fiber-rich diets (Aygun and Cerim, 2021), and the consumption of vegetables and fruits (Speirs et al., 2012; Soylar and Kadıoğlu, 2020). In contrast, adolescents with low health literacy were found to have a higher probability of having symptoms of eating disorders in Slovakia (Boberova and Husarova, 2021). To our knowledge, the current study is the first to explore the association between IHL and diversified dietary behaviors among Chinese college students.

The phenomenon of IHL promoting healthy dietary behaviors is explained by the transtheoretical model (TTM), in which self-efficacy plays a mediator role in the effect of IHL on dietary behaviors. According to the TTM, individuals in the later stages of change toward adopting healthier behaviors exhibit greater self-efficacy and perceive more advantages of their changed behavior when compared to early stages, concurrent with a decrease in the level of perceived barriers (Gullette et al., 2009). Self-efficacy represents the extent of confidence in one’s capability to modify a specific behavior (Bandura et al., 1999). From one perspective, the current study indicated that individuals with higher IHL could make informed decisions, understand and choose healthy diets, and finally achieve the goal of improving their overall health. Further, these results also indicate that using a framework based on TTM, the goal of future conventional communication strategies is not to convince individuals to change their behavior, but to help them enter the later stage of change of TTM as early as possible to build their confidence in changing healthy dietary behaviors in timely manner, subsequently enabling them to form healthy dietary habits.

Consistent with previous research findings, this study found that individuals with higher monthly incomes were more likely have healthier dietary behaviors. When students live independently on campus, they usually become more self-sufficient and purchase food based on their own income or family support (Deliens et al., 2014); suitable support encourages such students to develop healthier dietary habits (Sotos-Prieto et al., 2015). Additionally, this study also found that individuals whose parents had a high level of education were more likely have healthier dietary behavior. This may be because when parents receive more education, their children likely have easier access to general and specific knowledge related to health, as well as better problem-solving skills, which can help them make wiser decisions regarding food choices (Hasson et al., 2018).

Although a significant positive association was found between general health literacy levels and healthier dietary behaviors, we further examined the association between different dimensions of IHL and dietary behaviors, finding that, in addition to the health awareness factor, there were significant positive associations between physical activity and nutrition factors, and healthier dietary behaviors. Further, there was a significant negative association between interpersonal relationships and dietary behavior (Supplementary Table S1). This shows that the nutrition factor was more important than physical activity factors in positively predicting dietary behavior. This result is easily explained because nutrition literacy, which is conceptualized as an important component of health literacy (Blitstein and Evans, 2007), is defined as the ability to acquire, process, and comprehend nutrition-related information, along with the skills to make appropriate nutritional choices (Silk et al., 2008), which have a critical influence on eating behaviors (Taylor et al., 2019; Depboylu et al., 2023). Regarding physical activity, the significance of such behavior has been emphasized as contributing to a healthful diet across different populations (Castro et al., 2020). A prior study confirmed that active individuals have greater control over their eating behavior because motivation influences the recognition and integration of healthy eating habits into their lives (Carraça et al., 2020). Peer-based strategies could explain the association between interpersonal relationships and dietary behavior. Taking into account that both men are keen on muscle building and women on shaping their body, developing a stronger connection with peers might contribute to college students adopting healthier dietary behaviors (Smolak et al., 2005; Eisenberg and Neumark-Sztainer, 2010).

There were several limitations to this study. First, due to the cross-sectional study design, we were unable to establish a causal association between IHL levels and healthy dietary behaviors. Secondly, recall bias is another limitation, which is attributable to the self-reported questionnaire. Future research should use precise measurements of dietary intake behaviors, such as a weighted food record, to minimize measurement bias. Third, dietary behaviors were self-reported in this study, most likely leading to biased results towards the null in the estimated associations. However, considering that college students have most of their meals in a university canteen where serving sizes are almost standardized in China, it is important to mention that previous research has shown that using a semi-quantitative food frequency questionnaire with fixed portions not only increases the response burden for participants but does not enhance the validity of the Food Frequency Questionnaire (Hunter et al., 1988). Fourth, chronic diseases could confound the association between IHL and dietary behavior. Prior studies found inverse associations that health literacy and chronic conditions such as diabetes (Cravo et al., 2023), cardiovascular diseases (Vieira et al., 2021) and depression (Hsu et al., 2020). Furthermore, a follow-up study indicated that individuals who maintaining a healthy dietary patterns have decreased likelihood of developing chronic diseases (Wang et al., 2023). However, we did not collect variables such as chronic diseases. This means that this association might have been overestimated.

## 5 Implications

The current study further strengthens the evidence that a higher level of IHL is associated with the possibility of exhibiting healthy dietary behaviors among college students. In clinical settings, health education experts or health care professionals often suggest their clients to improve or maintain healthy dietary behaviors to reduce morbidity. This evidence-based study suggests that IHL, which denotes obtaining, understanding, and utilizing health information and services, and making reasonable decisions, should receive more attention regarding improving or maintaining healthy dietary behaviors because such literacy can encourage college students to take an active approach to health. It is highly recommended that researchers conduct further studies to explore how to effectively enhance university students’ IHL levels. Therefore, we suggest that dynamic monitoring of college students’ IHL and healthy dietary behaviors should be conducted. Additionally, IHL training programs need to be conducted within universities to raise college students’ awareness of taking an active approach to health and improve their IHL level.

## 6 Conclusion

The findings indicated a positive relationship between IHL and dietary behavior, such that the higher the level of IHL among college students, the healthier the dietary behaviors they tended to adopt in their daily lives. These findings suggest the importance of developing stages of change-based educational interventions, which could assist individuals with limited IHL not only to acquire necessary health-related knowledge but also to strengthen their motivation to engage in healthy dietary behaviors. Future studies should employ longitudinal prospective or randomized controlled designs to establish a causal association between IHL and healthy dietary behaviors.

## Data availability statement

The original contributions presented in the study are included in the article/Supplementary material, further inquiries can be directed to the corresponding authors.

## Ethics statement

The studies involving humans were approved by Research Institute of Educational Economics and Administration of Shenyang Normal University. The studies were conducted in accordance with the local legislation and institutional requirements. The participants provided their written informed consent to participate in this study. Written informed consent was obtained from the individual(s) for the publication of any potentially identifiable images or data included in this article.

## Author contributions

CM: Writing – review & editing, Writing – original draft, Investigation, Formal analysis, Data curation, Conceptualization. ZR: Writing – review & editing, Writing – original draft, Software, Methodology, Investigation, Funding acquisition, Formal analysis, Data curation, Conceptualization. ZC: Writing – review & editing, Writing – original draft, Supervision, Methodology, Conceptualization. CL: Writing – review & editing, Writing – original draft, Supervision, Resources, Methodology, Investigation, Formal analysis, Conceptualization.
