# Supplementary material for: The association between interactive health literacy and dietary behaviors among Chinese college students: a large-scale cross-sectional study
Source: Front Psychol. 2024 May 30;15:1363885. doi: 10.3389/fpsyg.2024.1363885 (PMC11169687; doi:10.3389/fpsyg.2024.1363885)
Supplement: Supplementary file 1 [file Table_1.docx]

**Supplements**

Table S1 The associations between dimension of IHL and numbers of dietary behaviors（n=9）

| Each dimension | Wald | OR (95% CI) | P value |
| --- | --- | --- | --- |
| Nutrition | 12.302 | 1.085 (1.048, 1.123) | <0.001 |
| Physical activity | 4.251 | 1.069 (1.034, 1.105) | 0.039 |
| Interpersonal relationships | 7.361 | 0.943 (0.904, 0.984) | 0.007 |
| Health awareness | 0.687 | 1.011 (0.986, 1.036) | 0.407 |

P-values and OR (95%CI) were computed through a logistic regression model adjusted for sex, age (continuous variable), annual family income (<20000, 20000-35000, ＞35000 yuan), place of residence (town or rural), and father and mother’s education (primary and below, junior high, high school, or bachelor degree and above).
